# Cardiovascular effects associated with chimeric antigen receptor T cell therapy in cancer patients: A meta-analysis

**DOI:** 10.3389/fonc.2022.924208

**Published:** 2022-11-09

**Authors:** Li-Rong Chen, Ya-Jia Li, Zheng Zhang, Ping Wang, Tao Zhou, Kai Qian, Yu-Xin Fan, Yu Guo, Gong-Hao He, Lei Shen

**Affiliations:** ^1^ College of Pharmacy, Dali University, Dali, China; ^2^ Department of Clinical Pharmacy, 920th Hospital of Joint Logistics Support Force, Kunming, China; ^3^ Medical Engineering Section, The 306th Hospital of People’s Liberation Army (PLA), Beijing, China

**Keywords:** chimeric antigen receptor T cell, CAR-T cell therapy, cardiovascular toxicities, safety, hematologic malignancies, cancer patients, meta-analysis

## Abstract

**Background:**

Although numerous studies confirmed the marked efficacy of chimeric antigen receptor T cells (CAR-T cells) in many hematologic malignancies, severe cardiovascular toxicities remain to be a major obstacle when incorporating this technology. Furthermore, previous individual investigations regarding the cardiovascular toxicities of CAR-T cell therapy also reported controversial conclusions. Therefore, a meta-analysis was performed to further evaluate the impacts of CAR-T cell therapy on cardiovascular toxicities.

**Methods:**

The PubMed, Embase, Web of Science, and ClinicalTrials.gov databases were searched for eligible studies up to April 2022. All analyses were carried out using the R 4.1.0 software.

**Results:**

Eventually, 25 related studies consisting of 2,059 patients were enrolled in the current meta-analysis. We discovered that the pooled incidence rate of the all-cause mortality rate was 14.1% and that the pooled incidence rates of overall cardiovascular (CV) events and CV events with cytokine release syndrome (CRS) grade ≥ 2 were 25.6% and 14.2%, respectively. The pooled incidence of hypotension was 28.6%. Further analysis showed that the incidence rates of arrhythmias, cardiovascular dysfunction, heart failure (HF), CV deaths, acute coronary syndrome (ACS), cardiomyopathy, cardiac arrest, and other CV events were 19.2%, 8.0%, 5.3%, 1.8%, 2.5%, 2.9%, 1.3%, and 1.9%, respectively.

**Conclusion:**

Cancer patients treated with CAR-T cell therapy were at risk for cardiovascular toxicities, of which the most common cardiovascular events were arrhythmias, cardiovascular dysfunction, and heart failure. These findings would contribute to achieving more rational and individualized use of CAR-T cells in clinical treatment.

## 1 Introduction

In recent decades, immune-based therapies had made extraordinary strides in clinical trials, offering the possibility of achieving long-term remission and even a complete cure for cancer patients (1, 2). Chimeric antigen receptor (CAR)-based T cell adoptive immunotherapy, as a personalized targeted immunotherapy option, is now becoming more and more popular in the war against tumors (3, 4). CAR is a recombinant fusion protein that is engineered to recognize tumor-associated antigens resulting in the activation of T cells and the destruction of target cells (5). The first CAR designs aiming at combating cancer emerged in the late 1980s and early 1990s, but only recent years have seen significant clinical success (6, 7), especially in certain hematologic malignancies (8–11), which offered promising efficacy in patients with leukemia or lymphoma (12).

Although CAR-T cell therapy has achieved remarkable advances, it was frequently accompanied by significant toxicity and even associated with potentially fatal adverse events such as cytokine release syndrome (CRS) and immune effector cell-associated neurotoxicity syndrome (ICANS) (13, 14). Therefore, comprehensively clarifying the characteristics of each adverse event and systematically analyzing their association with CAR-T cell therapy would be critical for the improvement of the clinical efficacy of CAR-T cells. In this regard, a surge of studies investigated the adverse events of the CAR-T cell therapy, which, however, intensively focused on CRS and neurotoxicity with few studies paying attention to the rest of the adverse events such as graft-versus-host disease (GVHD), tumor lysis syndrome (TLS), and cardiovascular toxicities (15–17). Therefore, a comprehensive evaluation of the rest adverse events will further prevent or reduce the incidence rate of adverse events in patients, helping to achieve more rational and individualized use of CAR-T cells in clinical treatment.

Among the rest of the adverse events that were not systematically analyzed, cardiovascular toxicity was particularly noteworthy. According to previous studies, immunotherapy-related cardiovascular toxicities conferred a significant risk of morbidity and mortality, which eventually restricted further development and wide application of CAR-T cell therapy in clinical treatment (18, 19). However, despite that, a number of investigations that evaluated cardiovascular toxicities of CAR-T cell therapy, its precise extent of cardiovascular toxicities, and its characteristics remain poorly defined (20, 21). For instance, one study based on 126 patients receiving CAR-T cell therapy (the target antigens of these cells included CD19, CD22, and BCMA) reported 33 (26%) cardiovascular (CV) events, with heart failure (HF), acute coronary syndrome (ACS), and arrhythmias being the top 3 CV events (22). In contrast, in a recent evaluation of 90 patients receiving CAR-T cell therapy, 17 (19%) CV events were, reported and the most common CV event was arrhythmia, which was followed by myocarditis and HF (23). Therefore, great differences were frequently suggested regarding both the overall incidence and varied kinds of CV events in cancer patients after CAR-T cell therapy (24–29). Furthermore, most previous individual clinical studies regarding cardiovascular toxicities were performed based on relatively small samples, which might not be powered enough to precisely estimate the clinical outcomes. Hence, it is still necessary to comprehensively explore the impact of the incidence of CV events in cancer patients after CAR-T cell therapy in order to further improve our understanding of CAR-T cell immunotherapy-related cardiovascular toxicities and promote the rational application of CAR-T cells. However, no such investigation has been conducted as far as we know.

Based on this background, the present study performed a meta-analysis that synthesizes the results from all available studies to assess the cardiovascular toxicities and the cardiovascular safety of CAR-T cell therapy, trying to offer further information for its future clinical application and research.

## 2 Materials and methods

### 2.1 Search strategy

This meta-analysis adhered to the Preferred Reporting Items for Systematic Reviews and Meta-Analyses (PRISMA) guidelines. A systematic search of the literature was conducted to identify published studies on all-cause mortality, cardiovascular toxicity, and hypotension related to CAR-T cell therapy. The PubMed, Embase, Web of Science, and ClinicalTrials.gov databases were searched for eligible studies. The search time was limited until 1 April 2022. The search terms “chimeric antigen receptor T cell”, “CAR-T”, and “Cancer” were used in the process of search. The search strategies for PubMed are provided in **Supplementary Table S1**. All studies related to the topics were screened. Additionally, we thoroughly searched the reference lists of related reviews and included articles to obtain potential investigations. When several publications on the same study population were included, only the most recent or complete study was used in this meta-analysis. The literature retrieval was conducted by two independent authors.

### 2.2 Inclusion and exclusion criteria

The inclusion criteria were as follows: 1) studies investigating cardiovascular effects of CAR-T cell therapy in cancer patients; 2) clinical trials, randomized or non-randomized controlled trials, and single-arm studies; 3) studies published in English; 4) the study participants were all patients with cancer treated with CAR-T cells and qualified studies reported at least one of the following outcomes: all-cause mortality, CV events, arrhythmias, cardiovascular dysfunction, HF, CV deaths, ACS, cardiomyopathy, cardiac arrest, other CV events, and hypotension. We excluded the following studies: 1) case series involving less than four patients; 2) reviews, editorials, animal experiments, and meta-analyses; 3) duplicated publications; 4) the study not reporting any of the following outcomes: all-cause mortality, CV events, arrhythmias, cardiovascular dysfunction, HF, CV deaths, ACS, cardiomyopathy, cardiac arrest, other CV events, and hypotension; 5) cancer patients with concurrent use of other anticancer interventions.

### 2.3 Data extraction

The data of the initial review were recorded on a standard data extraction form by two authors independently. If there were different judgments between these two researchers, discrepancies were settled by discussion or by adjudication by a third investigator. Extracted data included the following information: the name of the first author, publication year, the types of cancers, number of patients, gender, age, country of participants, target antigen of CAR-T cells, study type, and outcomes of interest, including all-cause mortality, CV events (including arrhythmias, cardiovascular dysfunction [including cardiac dysfunction, systolic dysfunction, and left ventricular systolic dysfunction], HF [including decompensated heart failure and chronic heart failure], CV deaths, ACS, cardiomyopathy, cardiac arrest, and other CV events), CV events (CRS grade ≥ 2) (defined as the incidence of cancer patients with both CV events and CRS grade ≥ 2 cytokine release syndrome among total cancer patients receiving CAR-T cell therapy), and hypotension.

### 2.4 Study qualitative assessment

As randomized controlled trial (RCT) and non-randomized studies were included in the present meta-analysis, we evaluated the quality of studies with the Cochrane Collaboration tool and Newcastle–Ottawa Quality Assessment Scale (NOS), respectively. The risk of bias in RCT studies was assessed by the Cochrane Collaboration tool, which includes sequence generation, allocation sequence concealment, blinding, missing outcome data, and other biases (30). In addition, the risk of bias in non-randomized studies was assessed by the NOS, which is a star system ranging from 0 to 9 stars. With the use of this “star system”, each included study was judged on three broad perspectives: the selection of the study groups, the comparability of the groups, and the ascertainment of the outcome of interest (31). High-quality studies were identified with a NOS score of 5 or more, whereas those with less than a score of 5 were considered low-quality studies. The qualitative assessment of the study was independently performed by two investigators, and any disagreements between the two investigators were resolved through discussion or by asking a third investigator.

### 2.5 Ethical statement

All results and analyses were based on previous ethically approved studies; thus, no further ethics approval and patient consent were required.

### 2.6 Statistical analysis

All statistical analyses were performed using R software version 4.1.0 with meta and metafor packages. Dichotomous data with 95% confidence intervals (95% CIs) were analyzed to estimate the cardiovascular toxicity of CAR-T cell therapy in cancer patients, and p ≤ 0.05 was considered statistically significant. Cochran’s Q test and Higgins’s I^2^ statistics were used to make heterogeneity tests for eligible studies. A random-effects model was used for the analysis when heterogeneity was significant (p ≤ 0.05 or I^2^ ≥ 50%). Otherwise, a fixed-effects model was employed. In addition, a subgroup analysis was conducted as well according to the target antigens of CAR-T cells. Meanwhile, a sensitivity analysis was performed to explore the source of heterogeneity and to assess the stability of the results by excluding each study successively. Moreover, we assessed publication bias by contour-enhanced funnel plots coupled with Egger’s test. When a symmetrical inverted funnel shape arose or Egger’s test yielded p-values greater than 0.05, we consider no publication bias.

## 3 Results

### 3.1 Study selection and qualitative assessment

**Figure 1:**
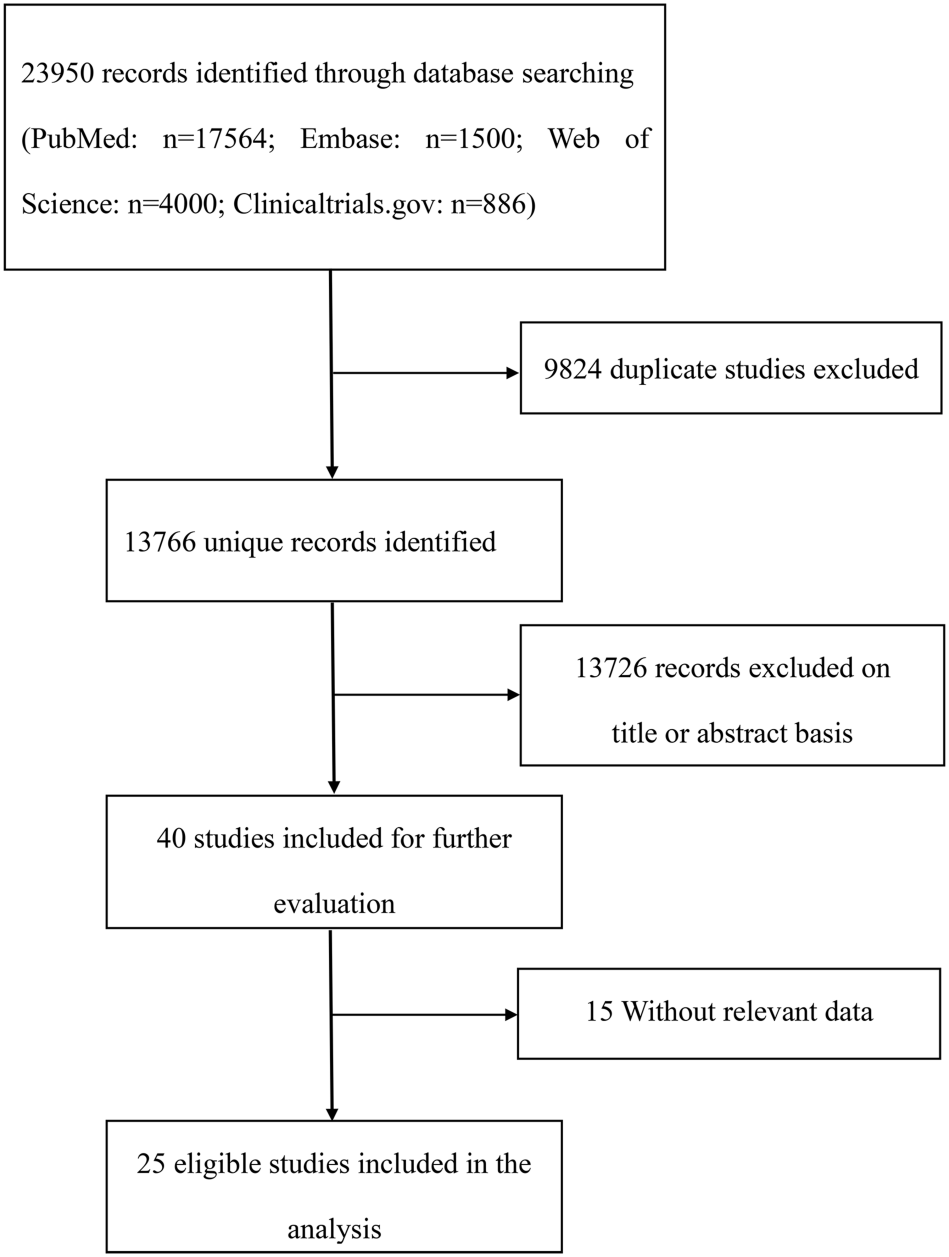
Flow diagram of the study selection process.

The flowchart illustrating the literature search process is shown in **Figure 1**. According to the literature search strategy, 13,766 probably pertinent studies were found after excluding 9,824 duplicating studies. Furthermore, after excluding 13,726 unrelated articles by screening the title and abstract, the remaining studies were further reviewed of the full texts, and 15 studies were excluded, which did not provide available data. Eventually, 25 studies were included according to the present inclusion and exclusion criteria. Seven RCTs and 18 retrospective studies with a total of 2,059 patients were included in this meta-analysis. The detailed characteristics of the included studies are shown in **Table 1**. The publications were from 2015 to 2022, most of which were conducted in the USA. The involved cancer types of the present study were mainly hematologic malignancies, including multiple myeloma (MM), acute lymphoblastic leukemia (ALL), diffuse large B-cell lymphomas (DLBCL), chronic lymphocytic leukemia (CLL), non-Hodgkin’s lymphoma (NHL), and primary mediastinal large B-cell lymphoma (PMBCL). However, no eligible studies that focused on the cardiovascular effects of CAR-T cell therapy in solid tumor patients were currently retrieved. The target antigens of CAR-T cells included CD19, BCMA, and others. Seven RCT studies were independently assessed for quality by using the Cochrane Collaboration tool, and 18 studies were independently assessed for quality by using NOS (cohort studies) system. The main limitations for included RCT studies were possible lack of random sequence generation and lack of allocation sequence concealment (**Supplementary Figure S1**). The overall cohort study quality was rated as moderate to high. However, since included cohort studies were single-arm studies, the NOS evaluation was not applicable to the selection of the non-exposed cohort (**Supplementary Table S2**).

**Table 1:** Characteristics of the included studies and participants.

First author	Cancer type (%)	Patients evaluated n (% male)	Median age (years)	Country	Target antigen	Study type (NCT number)	NOS score
Ganatra et al. (20)	R/R ANHL, DLBCL (73%)	116 (61%)	63 (19–80)	USA	CD19	Cohort	7
Shalabi et al. (21)	ALL, NHL	52 (78.8%)	13.4 (4.2–30.3)	USA	Others	Cohort	6
Qi etal. (22)	MM, lymphoma, ALL	126 (58%)	56 (6–72)	China	Others	Cohort	5
Brammer et al. (23)	R/R lymphomas	90 (57.8%)	61*	USA	Others	Cohort	8
Fitzgerald et al. (24)	R/R ALL	39 (NA)	11 (5–22)	USA	CD19	RCT (NCT01626495)	NA
Schuster et al. (25)	NHL, B-cell lymphomas	63 (63.5%)	65	USA	CD19	RCT (NCT02030834)	NA
Burstein et al. (26)	B-ALL (98%), T-ALL, B lymphoblastic lymphoma, PMBCL	98 (55%)	11.8 (1.7–27.1)	USA	CD19	Cohort	8
Locke et al. (27)	DLBCL, TFL, PMBCL, HGBCL	277 (65.34%)	65	USA	CD19	Cohort	8
Lefebvre et al. (28)	DLBCL (30%), ALL (25%), CLL (46%)	145 (74%)	60 (50–66)	USA	CD19	Cohort	6
Alvi et al. (29)	Lymphoma (88%), myeloma (8%)	137 (68%)	62 (54–70)	USA	Others	Cohort	7
Alvi et al. (32)	DLBCL (80%), MM (13%), other cancers (7%)	82 (69%)	60 ± 11*	USA	Others	Cohort	6
Brammer et al. (33)	Lymphoid malignancies	66 (62%)	60 (23–80)	USA	CD19	Cohort	7
Dalal et al. (34)	relapsed and refractory B-cell malignancies	75 (NA)	64	USA	Others	Cohort	6
Lee et al. (35)	R/R ALL or NHL	21 (66.70%)	1–30#	USA	CD19	RCT (NCT01593696)	NA
Lee et al. (36)	NHL	47 (NA)	NA	NA	CD19	Cohort	5
Lee et al. (37)	RRMM	22 (NA)	NA	NA	BCMA	Cohort	6
Lefebvre et al. (38)	ALL (7%), CLL (56%), DLBCL (38%)	90 (78%)	61 ± 10*	NA	Others	Cohort	7
Maude et al. (39)	R/R BCALL	75 (NA)	11 (3–23)	Multicenter	CD19	RCT (NCT02435849)	NA
NCT01029366 (2019)	CLL, ALL	20 (85%)	65	USA	CD19	RCT (NCT01029366)	NA
NCT01626495 (2020)	B-cell leukemia	73 (54.8%)	65	USA	CD19	RCT (NCT01626495)	NA
Neelapu et al. (40)	DLBCL (76%), PMBCL, or TFL (24%)	101 (67%)	58 (23–76)	USA, Israel	CD19	RCT (NCT02348216)	NA
Patel et al. (41)	Lymphoid malignancies	49 (NA)	68*	USA	CD19	Cohort	6
Patel et al. (42)	NA	75 (NA)	Adult patients	NA	Others	Cohort	8
Rothberg et al. (43)	DLBCL (80%), ALL (15%), other cancers (5%)	60 (60%)	54.94 ( ± 19)*	USA	Others	Cohort	7
Wudhikarn et al. (44)	Aggressive B-NHL	60 (70%)	62.9 (19.5–85.9)	USA	CD19	Cohort	7

R/R ANHL, refractory or relapsed, aggressive non-Hodgkin’s lymphoma; DLBCL, diffuse large B-cell lymphoma; ALL, acute lymphoblastic leukemia; NHL, non-Hodgkin’s lymphoma; MM, multiple myeloma; PMBCL, primary mediastinal B-cell lymphoma; TFL, transformed follicular lymphoma; HGBCL, high-grade B-cell lymphoma; CLL, chronic lymphocytic leukemia; R/R BCALL, relapsed or refractory B-cell acute lymphoblastic leukemia; NA, not available.

^#^Age range.

^*^Average age.

### 3.2 Meta-analysis

#### 3.2.1 Overall incidence of all-cause mortality

**Figure 2:**
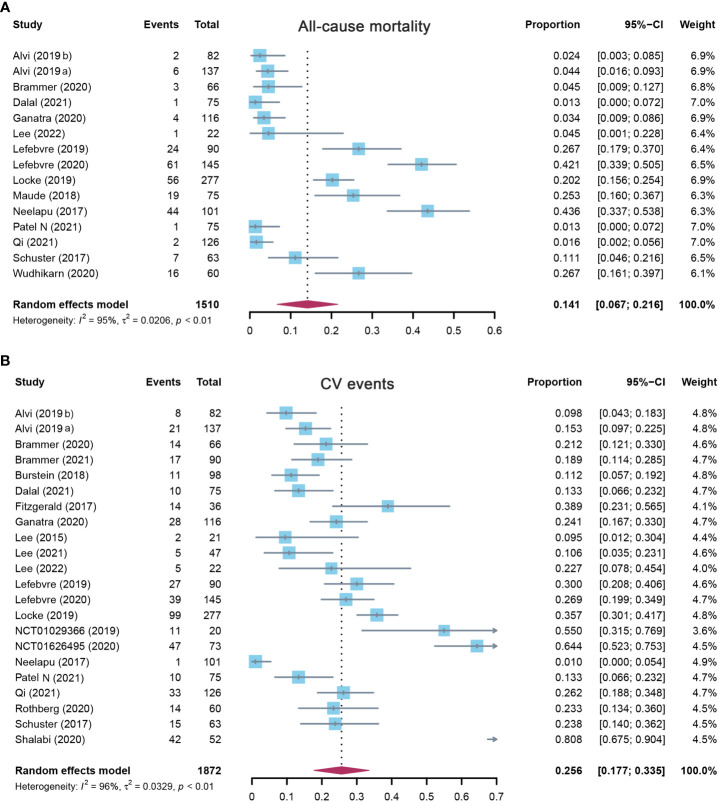
The forest plot of pooled incidence of **(A)** all-cause mortality and **(B)** CV event incidence rate in patients who received CAR-T cell therapy. CV, cardiovascular; CAR-T cell, chimeric antigen receptor T cell.

**Figure 3:**
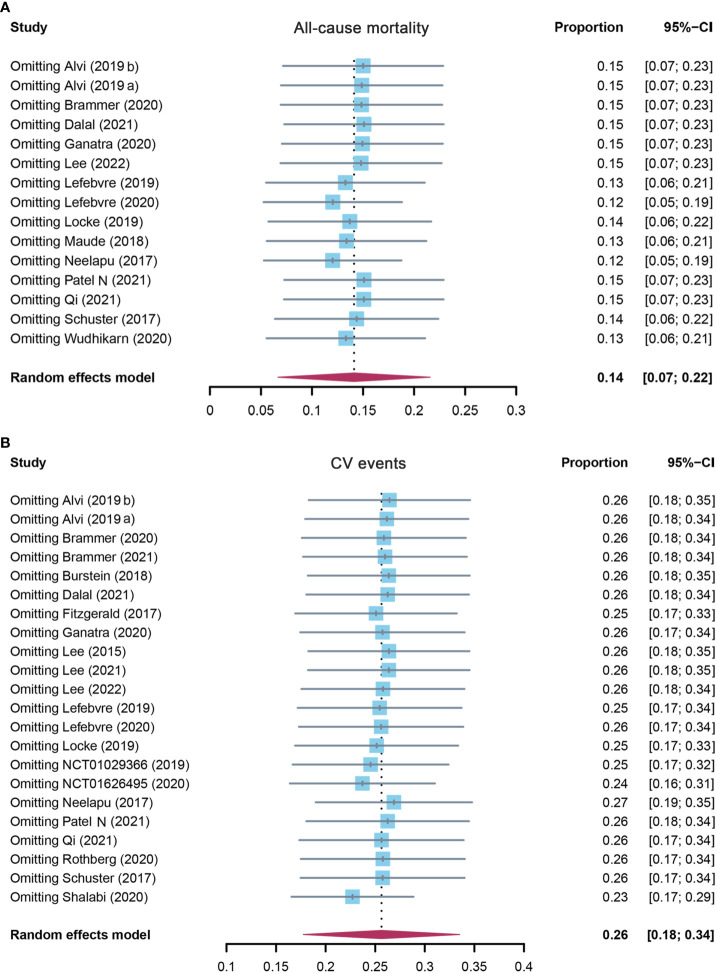
The sensitivity analysis of **(A)** all-cause mortality and **(B)** CV event incidence rate in patients who received CAR-T cell therapy. CV, cardiovascular; CAR-T cell, chimeric antigen receptor T cell.

Fifteen studies evaluated the overall incidence of all-cause mortality after CAR-T cell infusion in cancer patients. As shown in **Figure 2A**, the pooled proportion of all-cause mortality was 14.1% (95% CI: 0.067–0.216). As significant heterogeneity was observed (I^2^ = 95%, p < 0.01), a random-effects model was applied. In the subgroup analysis, all-cause mortality was also assessed according to different target antigens of CAR-T cells. The incidence rate of all-cause mortality was 21.7% (95% CI: 0.109–0.325) for the CD19 subgroup, 4.5% (95% CI: 0.001–0.228) for the BCMA subgroup, and 5.5% (95% CI: 0.000–0.123) for others (**Supplementary Table S3**). Furthermore, the sensitivity analysis was also performed by excluding each study successively. The sensitivity analysis observed that there was no study that greatly affected the result (**Figure 3A**), suggesting that the present result was stable.

#### 3.2.2 Overall incidence of cardiovascular events

A total of 22 studies assessed the overall incidence of CV events after CAR-T cell treatment in cancer patients. CV events were calculated as 25.6% (95% CI: 0.177–0.335) for all patients, and a random-effects model was used (I^2^ = 96%, p < 0.01) (**Figure 2B**). In the subgroup analysis, the incidence rate of CV events was 26.0% (95% CI: 0.156–0.365) for the CD19 subgroup, 22.7% (95% CI: 0.078–0.454) for the BCMA subgroup, and 25.4% (95% CI: 0.115–0.394) for others (**Supplementary Table S3**). The following sensitivity analysis observed that there was no study that greatly affected the result (**Figure 3B**).

#### 3.2.3 Incidence of arrhythmias

**Figure 4:**
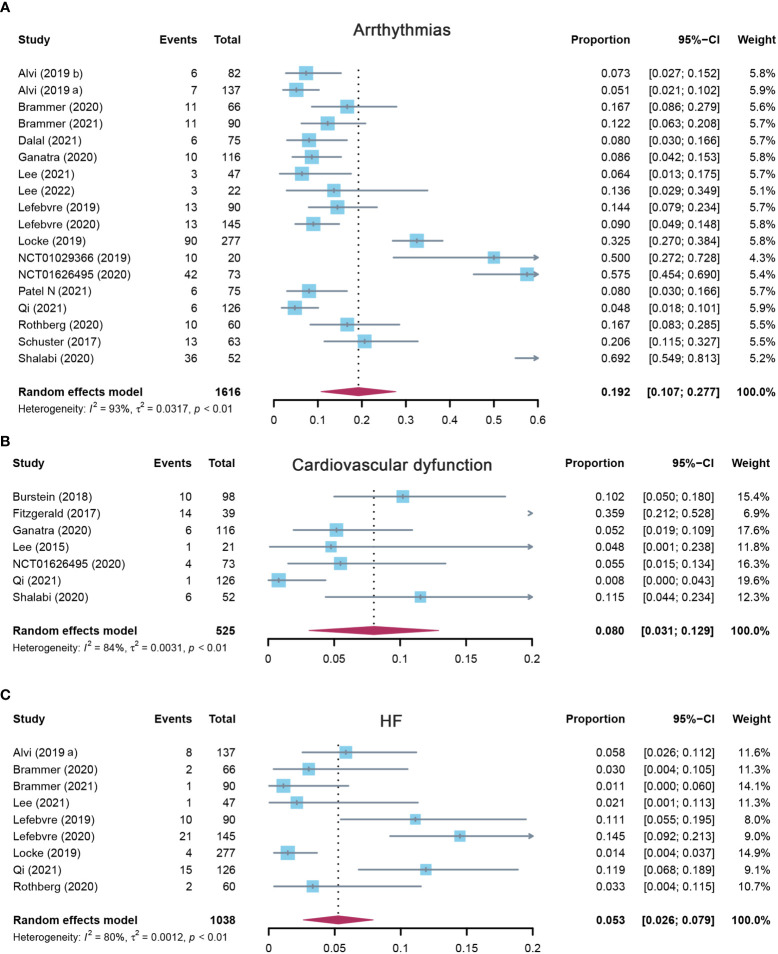
The forest plot of pooled incidence of **(A)** arrhythmias, **(B)** cardiovascular dysfunction, and **(C)** HF incidence rate in patients who received CAR-T cell therapy. HF, heart failure; CAR-T cell, chimeric antigen receptor T cell.

**Figure 5:**
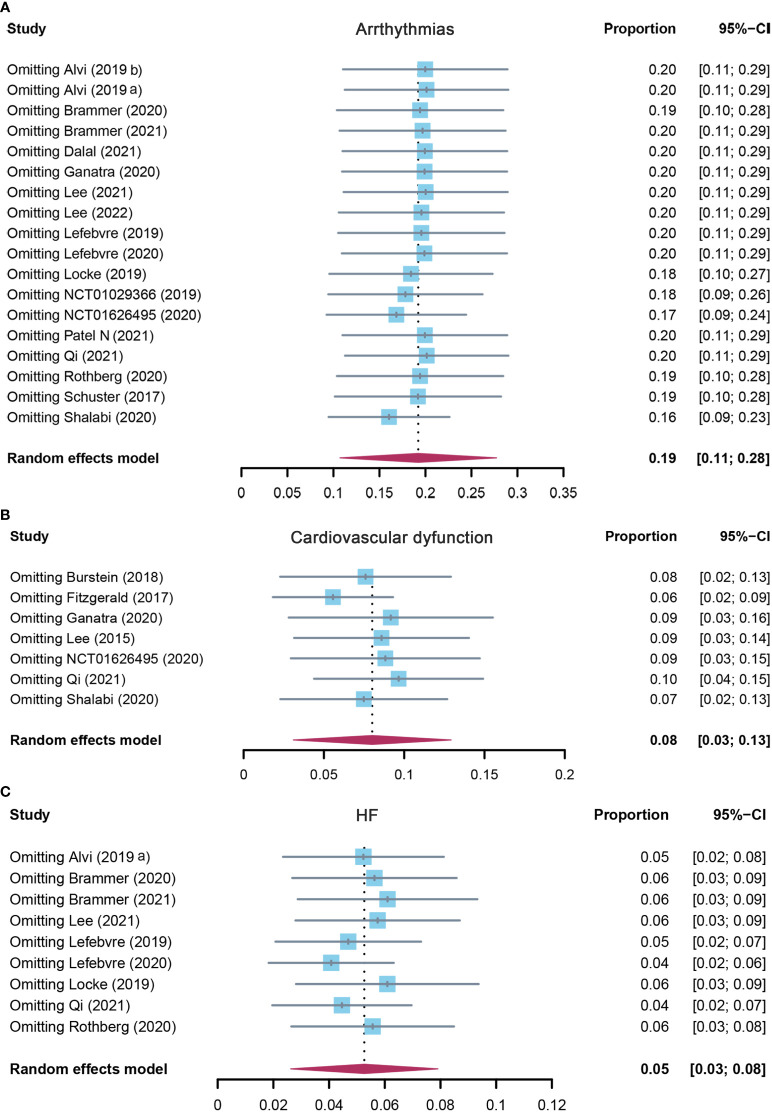
The sensitivity analysis of **(A)** arrhythmias, **(B)** cardiovascular dysfunction, and **(C)** HF incidence rate in patients who received CAR-T cell therapy. HF, heart failure; CAR-T cell, chimeric antigen receptor T cell.

Eighteen studies reported arrhythmias, and the pooled incidence of arrhythmias was 19.2% (95% CI: 0.107–0.277). There was significant heterogeneity among the included studies, and a random-effects model was used (I^2^ = 93%, p < 0.01) (**Figure 4A**). In the subgroup analysis, the incidence rate of arrhythmias was 24.1% (95% CI: 0.110–0.372) for the CD19 subgroup, 13.6% (95% CI: 0.029–0.349) for the BCMA subgroup, and 19.2% (95% CI: 0.107–0.277) for others (**Supplementary Table S3**). The sensitivity analysis showed that none of the studies had significantly interfered with the results of this meta-analysis (**Figure 5A**).

#### 3.2.4 Incidence of cardiovascular dysfunction

Seven studies evaluated the incidence of cardiovascular dysfunction after CAR-T cell infusion in cancer patients. The pooled proportion of cardiovascular dysfunction was 8.0% (95% CI: 0.031–0.129). There was significant heterogeneity among the included studies (I^2^ = 84%, p < 0.01), and a random-effects model was used (**Figure 4B**). In the subgroup analysis, the incidence rate of cardiovascular dysfunction was 9.8% (95% CI: 0.043–0.225) for the CD19 subgroup and 3.5% (95% CI: 0.003–0.479) for others (**Supplementary Table S3**). The sensitivity analysis showed that none of the studies had significantly interfered with the results of this meta-analysis (**Figure 5B**).

#### 3.2.5 Incidence of heart failure

A total of nine studies assessed the incidence of HF after CAR-T cell therapy in cancer patients. The pooled proportion of HF was 5.3% (95% CI: 0.026–0.079). There was significant heterogeneity among the included studies (I^2^ = 80%, p < 0.01), and a random-effects model was used (**Figure 4C**). In the subgroup analysis, the incidence rate of HF was 3.9% (95% CI: 0.012–0.125) for the CD19 subgroup and 7.1% (95% CI: 0.040–0.126) for others (**Supplementary Table S3**). The sensitivity analysis observed that there was no study that greatly affects the result of this meta-analysis (**Figure 5C**).

#### 3.2.6 Overall incidence of cardiovascular deaths

There were six studies that reported the overall incidence of CV deaths after CAR-T cell therapy in cancer patients. The pooled proportion of CV deaths was 1.8% (95% CI: 0.008–0.028), and a fixed-effects model was used (I^2^ = 0%, p = 0.73) (**Supplementary Figure S2A**). In the subgroup analysis, the incidence rate of CV deaths was 1.4% (95% CI: 0.002–0.049) for the CD19 subgroup and 1.9% (95% CI: 0.007–0.032) for others (**Supplementary Table S3**). The sensitivity analysis was still conducted and also did not influence the present result (**Supplementary Figure S3A**), further confirming that the present meta-analysis result is robust and credible.

#### 3.2.7 Incidence of acute coronary syndrome

Four studies assessed the incidence of ACS after CAR-T cell therapy in cancer patients. The pooled proportion of ACS was 2.5% (95% CI: 0.010–0.040). There was no significant heterogeneity among the included studies (I^2^ = 48%, p = 0.12), and a fixed-effects model was used (**Supplementary Figure S2B**). In the subgroup analysis, the incidence rate of ACS was 1.4% (95% CI: 0.002–0.049) for the CD19 subgroup and 4.4% (95% CI: 0.020–0.069) for others (**Supplementary Table S3**). The sensitivity analysis showed that none of the studies significantly affected the result of these pooled data (**Supplementary Figure S3B**).

#### 3.2.8 Incidence of cardiomyopathy

There were seven studies that reported the overall incidence of cardiomyopathy after CAR-T cell therapy in cancer patients. The pooled proportion of cardiomyopathy was 2.9% (95% CI: 0.005–0.054), and a random-effects model was used (I^2^ = 68%, p < 0.01) (**Supplementary Figure S2C**). In the subgroup analysis, the incidence rate of cardiomyopathy was 2.5% (95% CI: 0.000–0.063) for the CD19 subgroup, 9.1% (95% CI: 0.011–0.292) for the BCMA subgroup, and 4.0% (95% CI: 0.009–0.071) for others (**Supplementary Table S3**). The sensitivity analysis was still conducted and also did not influence the present result (**Supplementary Figure S3C**).

#### 3.2.9 Incidence of cardiac arrest

A total of six studies assessed the incidence of cardiac arrest after CAR-T cell therapy in cancer patients. The pooled proportion of cardiac arrest was 1.3% (95% CI: 0.004–0.022). There was no heterogeneity among the included studies (I^2^ = 0%, p = 0.98), and a fixed-effects model was used (**Supplementary Figure S2D**). The sensitivity analysis was still conducted and also did not influence the present result (**Supplementary Figure S3D**).

#### 3.2.10 Incidence of other cardiovascular events

A total of five studies assessed the incidence of other CV events after CAR-T cell therapy in cancer patients. The pooled proportion of other CV events was 1.9% (95% CI: 0.004–0.034). There was no significant heterogeneity among the included studies (I^2^ = 0%, p = 0.51), and a fixed-effects model was used (**Supplementary Figure S2E**). In the subgroup analysis, the incidence rate of other CV events was 1.9% (95% CI: 0.000–0.042) for the CD19 subgroup and 2.8% (95% CI: 95% CI: 0.000–0.071) for others (**Supplementary Table S3**). The sensitivity analysis showed that none of the studies significantly affected the result of these pooled data (**Supplementary Figure S3E**).

#### 3.2.11 Incidence of cardiovascular events (cytokine release syndrome grade ≥ 2)

Next, we further studied indirect toxicities related to CRS. Four studies further assessed the incidence of CV events (CRS grade ≥ 2) after CAR-T cell therapy in cancer patients. The pooled proportion of CV events (CRS grade ≥ 2) was 14.2% (95% CI: 0.112–0.179) (**Supplementary Figure S4A**) based on a fixed-effects model (I^2^ = 22%, p = 0.28). Furthermore, the pooled incidence rate of CRS among patients with CV events was 87.5% (95% CI: 0.696–1.000) (**Supplementary Figure S5**). In the subgroup analysis, the incidence rate of CV events (CRS grade ≥ 2) was 9.5% (95% CI: 0.048–0.163) for the CD19 subgroup and 15.4% (95% CI: 0.119–0.200) for others (**Supplementary Table S3**). The sensitivity analysis observed that there was no study that greatly affects the result of this meta-analysis (**Supplementary Figure S6A**).

#### 3.2.12 Incidence of hypotension

Since hypotension was also suggested to be an important outcome other than traditional CV events (21), we further evaluated its incidence. Thirteen studies assessed the incidence of hypotension after CAR-T cell treatment in cancer patients. The pooled proportion of hypotension was 28.6% (95% CI: 0.158–0.414). There was significant heterogeneity among the included studies (I^2^ = 96%, p < 0.01), and a random-effects model was used (**Supplementary Figure S4B**). In the subgroup analysis, the incidence rate of hypotension was 31.5% (95% CI: 0.169–0.461) for the CD19 subgroup and 18.4% (95% CI: 0.132–0.235) for others (**Supplementary Table S3**). The sensitivity analysis observed that there was no study that greatly affected the result (**Supplementary Figure S6B**).

### 3.3 Analysis of publication bias

The risk of publication bias was evaluated by funnel plot and Egger’s tests (**Supplementary Figure S7** and **Table S4**). No evidence of potential publication bias was revealed for outcomes such as CV deaths, cardiac arrest, other CV events (CRS grade ≥ 2), and hypotension according to Egger’s tests (p > 0.05) (**Supplementary Table S4**). However, publication bias was identified by Egger’s tests (p < 0.05) regarding the outcomes of all-cause mortality, CV events, arrhythmias, cardiovascular dysfunction, HF, ACS, and cardiomyopathy **(Supplementary Table S4**).

## 4 Discussion

In current clinical practice, cardiovascular toxicities remain a major risk when incorporating CAR-T cell therapy technology (45). To our knowledge, this is the largest and most comprehensive meta-analysis of treatment-related cardiovascular toxicities observed and encountered in CAR-T cell therapy. In the present study, the type of the neoplasm mainly focused on ALL, DLBCL, and lymphomas (ClinicalTrials.gov number: NCT01029366, NCT02348216, NCT02030834, etc.). The results showed that cancer patients treated with CAR-T cell therapy were at risk for cardiovascular toxicities, of which the most common cardiovascular events were arrhythmias, cardiovascular dysfunction, and HF. Furthermore, the subgroup analyses according to target antigens of CAR-T cells illustrated that the CV events exhibited the highest incidence in the CD19 subgroup, followed by others and the BCMA subgroup. However, since most current original studies had focused on CD19, the incidence of the CD19 subgroup in the present study might be exaggerated and should therefore be interpreted with caution. In this regard, further research is necessary to elucidate which target antigens of CAR-T cells has a higher risk of CV events. In general, our results further systematically confirmed the recent individual findings regarding the cardiovascular adverse effect of CAR-T cell therapy and may be helpful to develop novel CAR technologies to decrease their cardiovascular toxicity.

As CAR-T cell use expands, it becomes imperative to truly understand the mechanism behind cardiovascular injury, as this might help in the early intervention and prevention of cardiotoxicity. Several potential mechanisms were reported for CAR-T cell-mediated toxicity, which also affected the cardiovascular system. One possible mechanism was “on-target off-tumor” toxicity. High-affinity CARs are unable to discriminate between tumor cells and healthy tissues at physiologic levels (46, 47). Thus, normal tissues with a certain expression of tumor-associated antigens can be mistargeted by the scFv (48). Another possible mechanism was “off-target off-tumor” toxicity. It was reported that certain genetically engineered T cells with affinity-enhanced T-cell receptors targeted MAGE-A3, which was an antigen widely expressed in melanoma and myeloma. Nevertheless, titin, a protein expressed in striated cardiac muscle tissue, also contains an epitope that is very similar to an epitope on MAGE-A3 (49). It was determined by histopathologic analysis that the acute cardiotoxicity was due to off-target cross-reactivity against titin (49). Presumably, CAR-T cells were also indicated to cross-react with certain proteins in normal tissues that are similar to the target antigen (50). However, this mechanism has not been directly observed in CAR-T cell therapy trials so far. Therefore, further research is still needed to validate this hypothesis.

**Figure 6:**
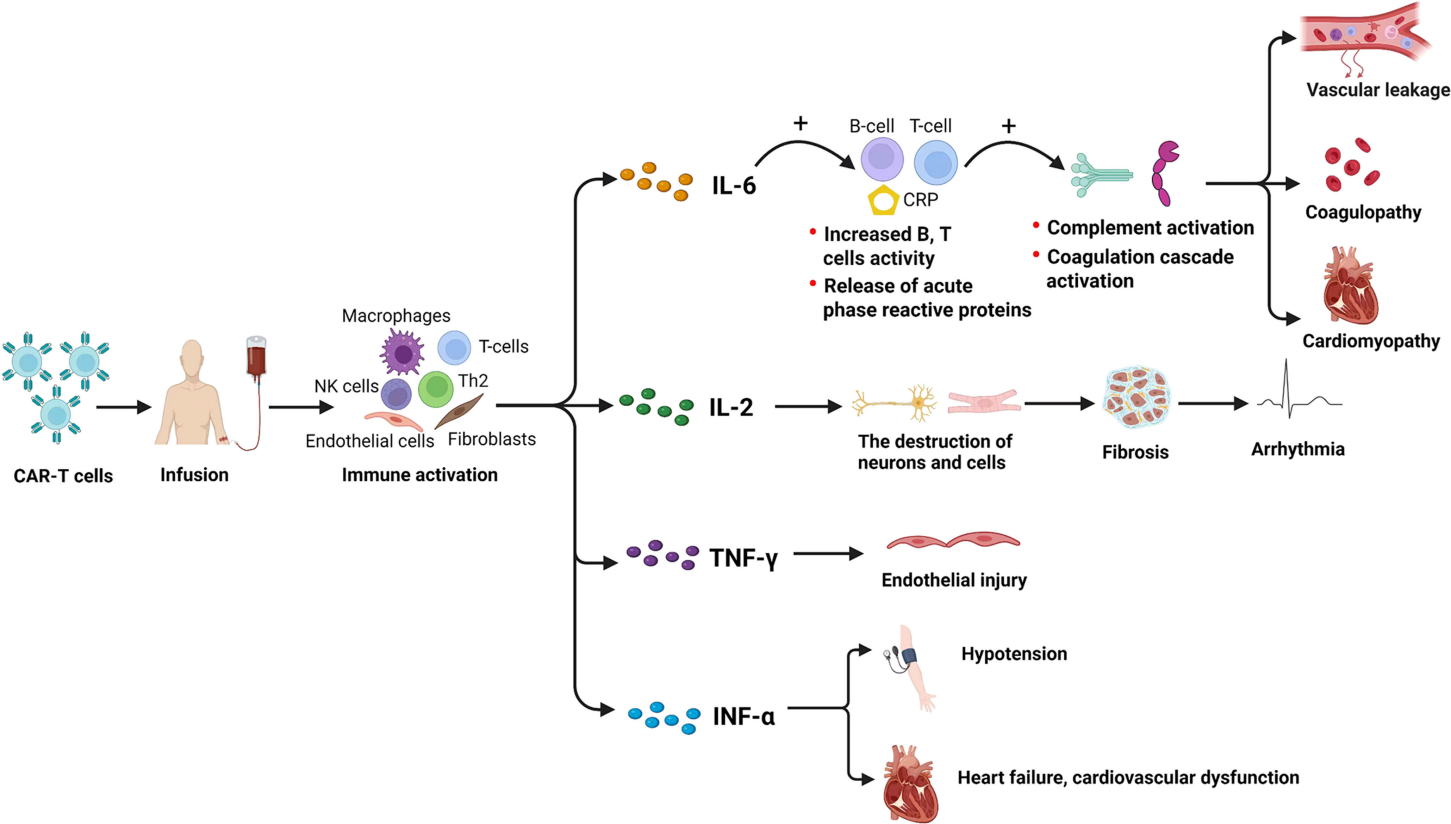
Potential cytokine-related mechanisms underlying the CV effects induced by CAR-T cell therapy. CV, cardiovascular; CAR-T cell, chimeric antigen receptor T cell. Created with BioRender.com.

In addition to the abovementioned possible direct effect, certain indirect cardiovascular effects of CAR-T cells were also reported, which included systemic inflammation (50) and cytokine-mediated effects (51). It was indicated that cardiovascular toxicity associated with CAR-T cell therapy might be caused by a release of a series of inflammation-related cytokines, such as IL-6, IL-2, TNF-α, and IFN-γ, from the infused CAR-T cells (14, 52). Previous studies reported that IL-6 is a primary driver of inflammation in the CAR-T cells activation pathway, leading to increased B- and T-cell activities and the release of acute phase reactive proteins (53). As a result, CAR-T cell-associated cardiotoxicity was usually accompanied by a marked rise in IL-6 (54), which contributed to complement and coagulation cascade activation and led to vascular leakage, coagulopathy, and cardiomyopathy (55). Moreover, it was also reported that IL-2 led to the destruction of cells and neurons, fibrosis, and blockage of the cardiac conduction system, which favors arrhythmia by predisposing to reentrant tachycardias (56), and that IFN-γ was related to increased release of severe cardiac disorders by causing endothelial injury (57, 58). In addition, TNF-α was also found to be highly associated with other cardiovascular events such as hypotension, HF, and cardiovascular dysfunction (59, 60) (**Figure 6**). Therefore, IL-6 and other cytokines might be key mediators involved in the development of cardiovascular toxicity of CAR-T cell therapy. Since IL-6 receptor blocker has already been used in clinical as the first-line treatment of cardiovascular toxicity after CAR-T cell infusion (51, 61, 62), other blockers or neutralizing antibodies that target IL-2, TNF-α, or IFN-γ, etc., would also be a promising treatment against the cardiovascular toxicity of CAR-T cell therapy and are worth being paid further attention to in future studies.

Moreover, according to the present study, prompt treatment strategies are also critical for reducing the severity of cardiovascular toxicity after CAR-T cell therapy, as the most common identified cardiovascular events in the present study (i.e., arrhythmias, cardiovascular dysfunction, and HF) are fatal. Although the specific treatment recommendations regarding this issue were not given by present guidelines, some relevant treatment strategies had already been widely applied by many centers in clinical practice. For instance, tocilizumab is usually used for the first-line therapy of cardiovascular toxicity, but corticosteroids were also used when patients do not respond to an initial dose of tocilizumab within 24 h (63), which were considered a part of first-line therapy in patients with acute life-threatening toxic effects (e.g., malignant dysrhythmias) (50). Additionally, if significant left ventricular dysfunction is diagnosed, patients may benefit from a substitution of vasopressor therapy for inotropic support with intravenous dobutamine therapy, while diuretic therapy may be used judiciously for relief of HF on dobutamine support (64). In this regard, our study may help clinicians pay more attention to cardiovascular toxicity after CAR-T cell therapy and will also contribute to providing a certain reference value for the development of novel treatment strategies and to the establishment of guidelines for prevention.

In addition to timely treatment strategies, early prevention measures are still worthy of attention. Pre-therapy evaluation often includes cardiovascular history and physical examination, functional assessment, electrocardiography, and echocardiography. Cardio-oncology review and stress testing are recommended when it is identified as a high cardiac risk profile (65). Clearly, close monitoring and early intervention of cardiovascular toxicities after the administration of CAR-T cell therapy are pivotal.

It should be mentioned that a certain degree of heterogeneity between the pooled studies was observed in the present study. Several factors may have contributed to this heterogeneity. First, cardiovascular toxicity is less exactly defined due to the paucity of data in the literature. Second, the follow-up time in the original studies was also different. The currently reported cardiotoxicities associated with CAR-T cell therapy were short-term effects, which were generally reversible. However, further subgroup regarding the follow-up time was not performed because of the deficiency of original data and relevant information. Third, CAR-T cell immunotherapy was less evaluated using RCTs, and numerous other confounding factors may hence lead to heterogeneity of the final outcomes. Therefore, large multicenter RCT studies are greatly warranted to verify our results and confirm the effect of cardiovascular toxicities of CAR-T cell therapy.

Several additional potential limitations should also be mentioned. First, the sample size is still relatively small and may not be powered to precisely estimate the clinical outcomes. More studies with larger sample sizes were hence suggested to offer a more representative analysis. Second, the subgroup analyses regarding age, ethnicity, type of cancer, tumor site, and CAR-T cell dose were not performed due to the lack of relevant data. Third, other potential confounding risk factors, such as significant disease burden, advanced age, multiple comorbidities, and prior exposure to cardiotoxins (e.g., anthracyclines, radiation, and tyrosine kinase inhibitors) (26, 66), were also unable to be adjusted currently, leading to a latent bias of the present results. Fourth, only published studies were included in the meta-analysis; some selection bias might possibly influence the reliability of our study results. Finally, our meta-analysis only focused on the publications from English databases, which might result in a potential language bias. These limitations should be noticed and addressed in future clinical investigations.

## 5 Conclusions

Our meta-analysis showed that cancer patients treated with CAR-T cell therapy were at risk for cardiovascular toxicities, of which the most common cardiovascular events were arrhythmias, cardiovascular dysfunction, and HF. Therefore, clinicians should pay more attention to the occurrence of these kinds of cardiotoxicity and provide prompt prevention and intervention to enhance the safety of CAR-T cell therapy. Furthermore, a deeper understanding of cardiotoxicity will help to promote the development of novel approaches to reduce toxicity and improve outcomes of CAR-T cell therapy.

## Data availability statement

The original contributions presented in the study are included in the article/**Supplementary Material**. Further inquiries can be directed to the corresponding authors.

## Author contributions

L-RC, Y-JL, ZZ, LS, and G-HH participated in the study design, conceptualization, and manuscript preparation. L-RC, Y-JL, PW, TZ, KQ, and Y-XF were responsible for the literature search and screening. ZZ and YG were responsible for data acquisition. L-RC, Y-JL, and ZZ were responsible for the quality assessment of included studies. L-RC, Y-JL, and ZZ were responsible for data analysis and interpretation of results. G-HH and LS were responsible for manuscript editing and review. All authors contributed to the article and approved the submitted version.

## Funding

This work was supported by grants from the National Science Foundation of China (No. 81960664), the Applied Basic Research Program of Yunnan Province of China (Joint Special Project of Kunming Medical University) (No. 202101AY070001-300), and the Applied Basic Research Program of Yunnan Province of China (No. 202101AT070030).

## Acknowledgments

We thank all authors for their contribution to this study.

## Conflict of interest

The authors declare that the research was conducted in the absence of any commercial or financial relationships that could be construed as a potential conflict of interest.

## Publisher’s note

All claims expressed in this article are solely those of the authors and do not necessarily represent those of their affiliated organizations, or those of the publisher, the editors and the reviewers. Any product that may be evaluated in this article, or claim that may be made by its manufacturer, is not guaranteed or endorsed by the publisher.
