# Supplementary figures and images for: Cardiovascular effects associated with chimeric antigen receptor T cell therapy in cancer patients: A meta-analysis
Source: Front Oncol. 2022 Nov 9;12:924208. doi: 10.3389/fonc.2022.924208 (PMC9682079; doi:10.3389/fonc.2022.924208)

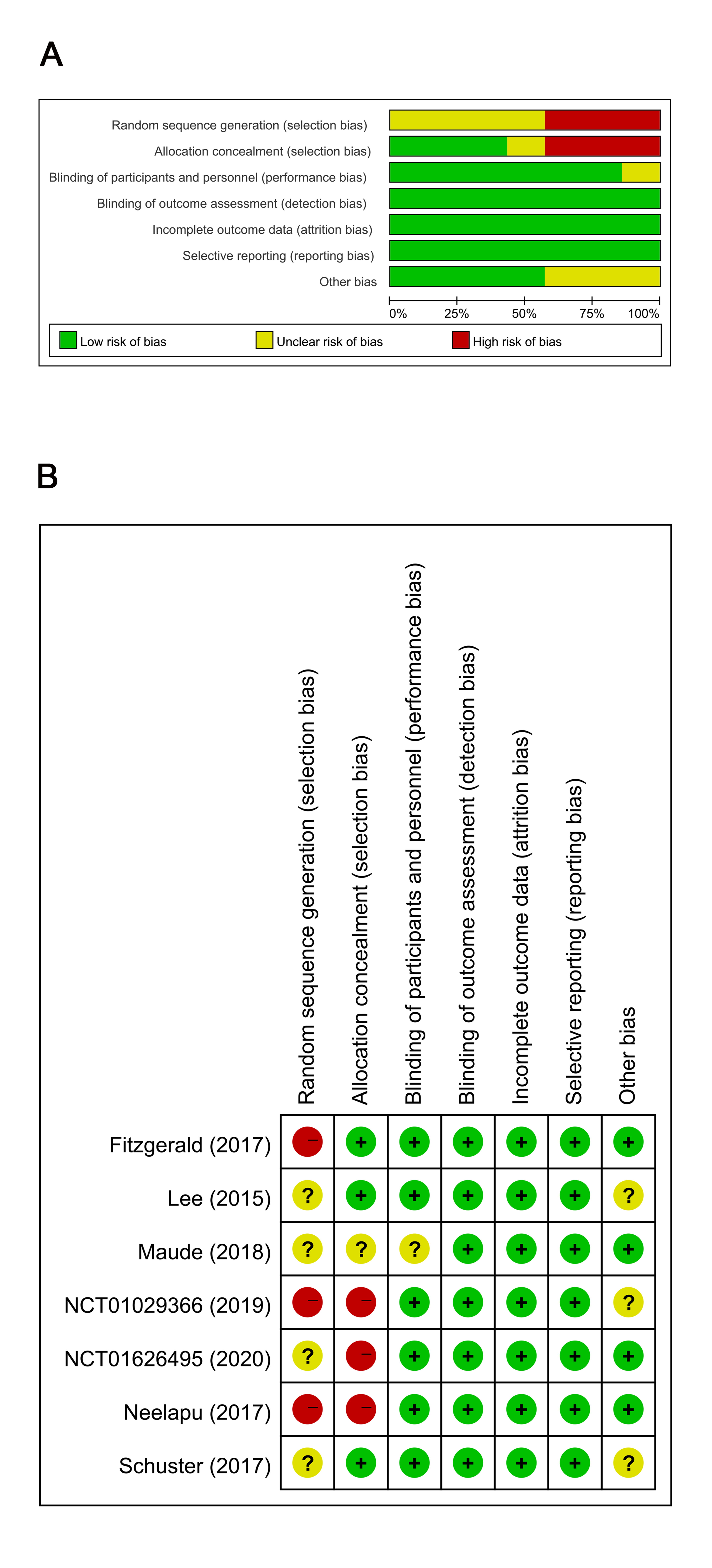

Supplement: Supplementary file 1 [file DataSheet_1.zip › Supplemental materials/Supplemental Figure S1.tif]

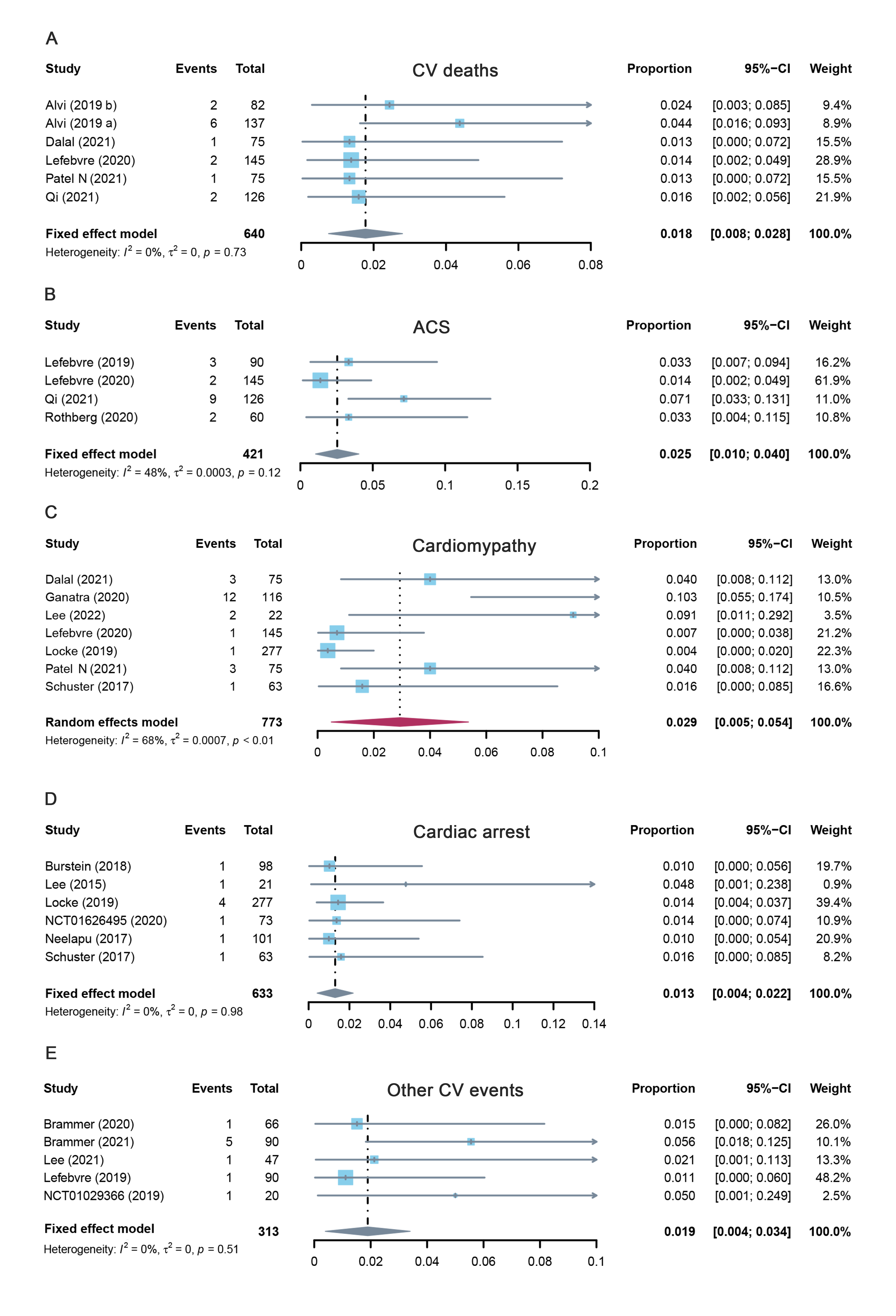

Supplement: Supplementary file 1 [file DataSheet_1.zip › Supplemental materials/Supplemental Figure S2.tif]

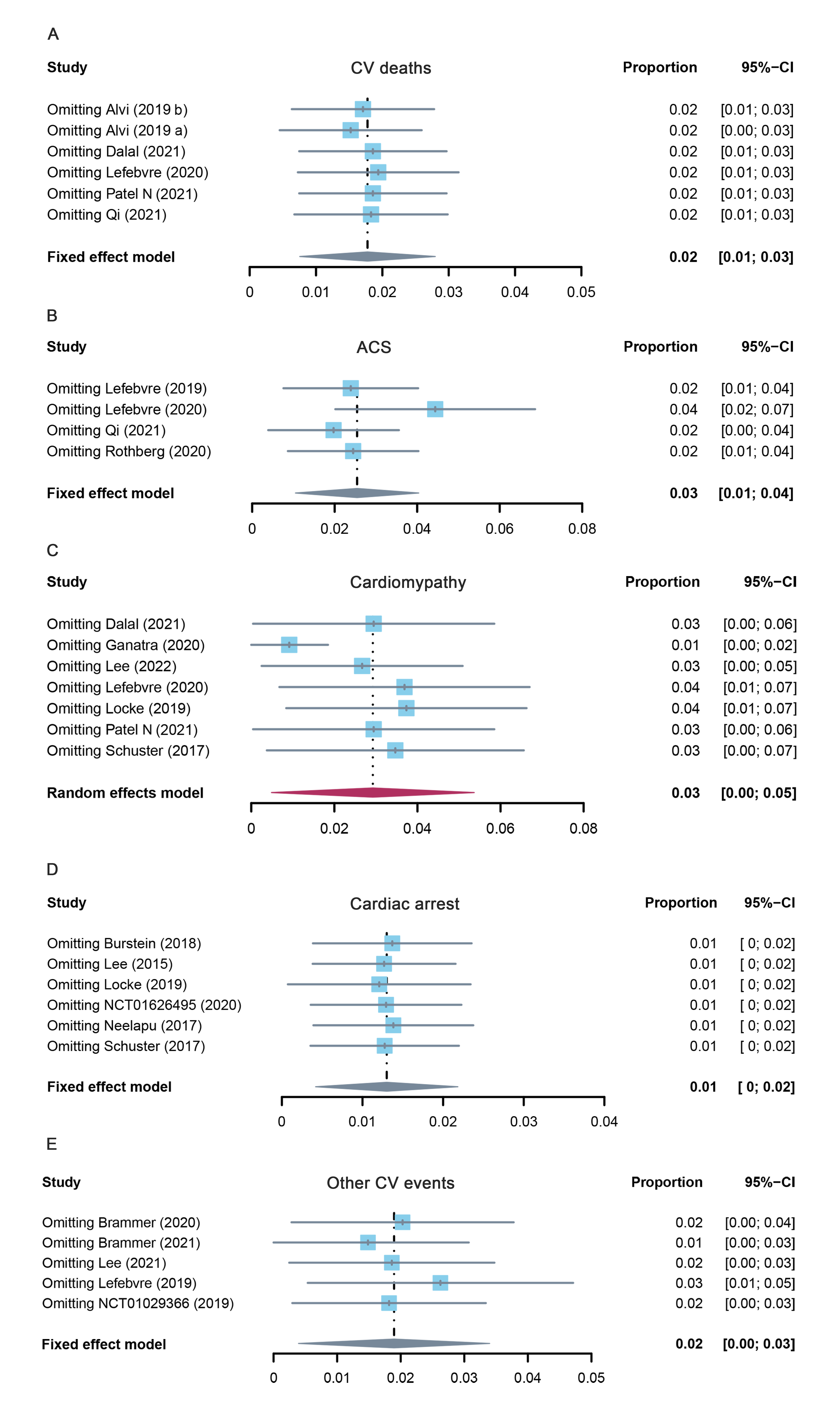

Supplement: Supplementary file 1 [file DataSheet_1.zip › Supplemental materials/Supplemental Figure S3.tif]

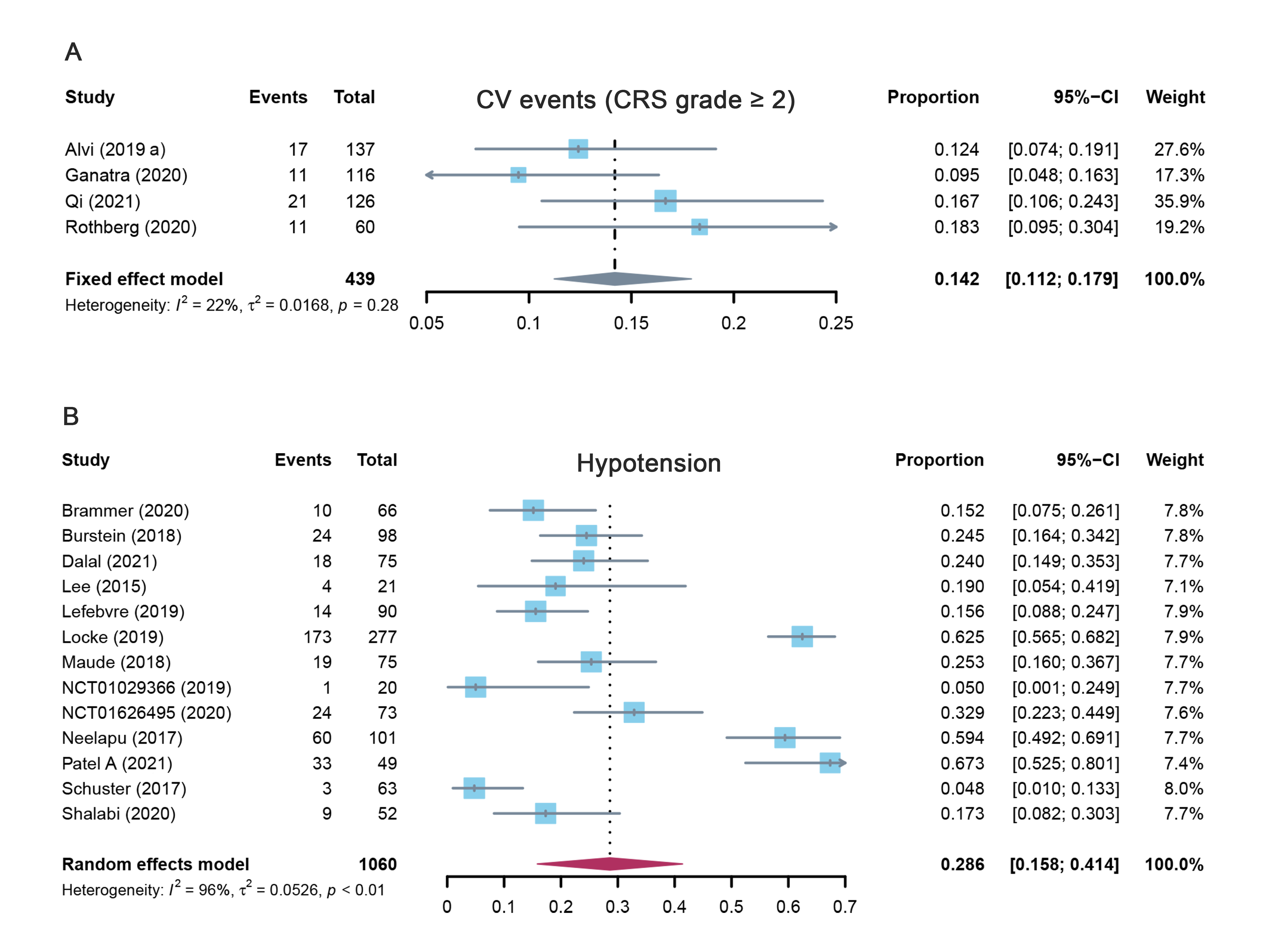

Supplement: Supplementary file 1 [file DataSheet_1.zip › Supplemental materials/Supplemental Figure S4.tif]

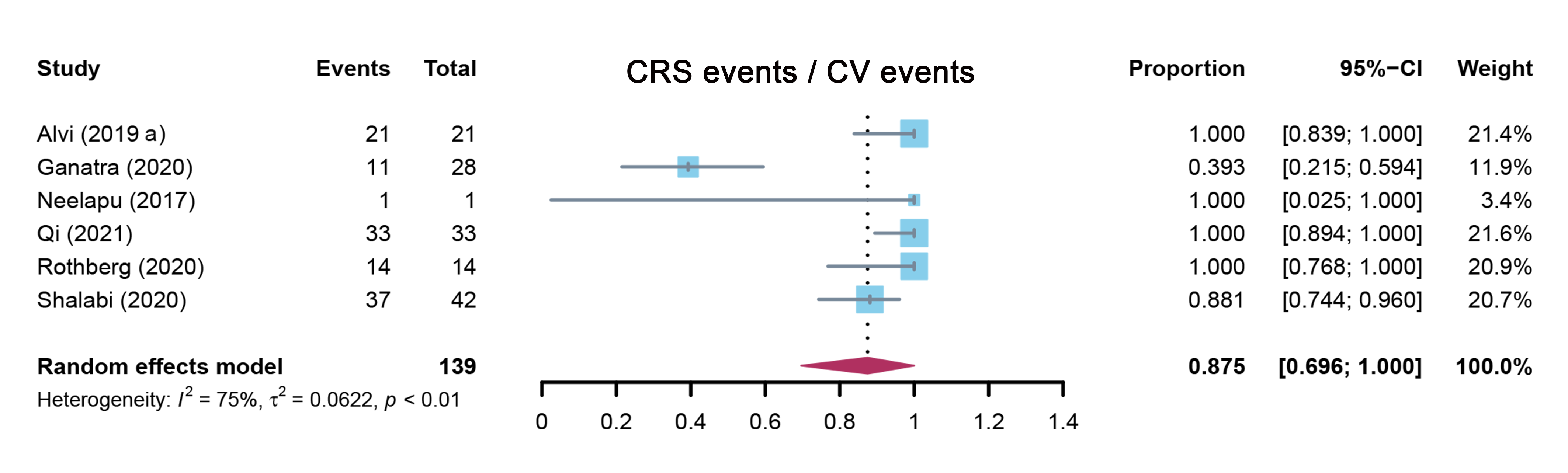

Supplement: Supplementary file 1 [file DataSheet_1.zip › Supplemental materials/Supplemental Figure S5.tif]

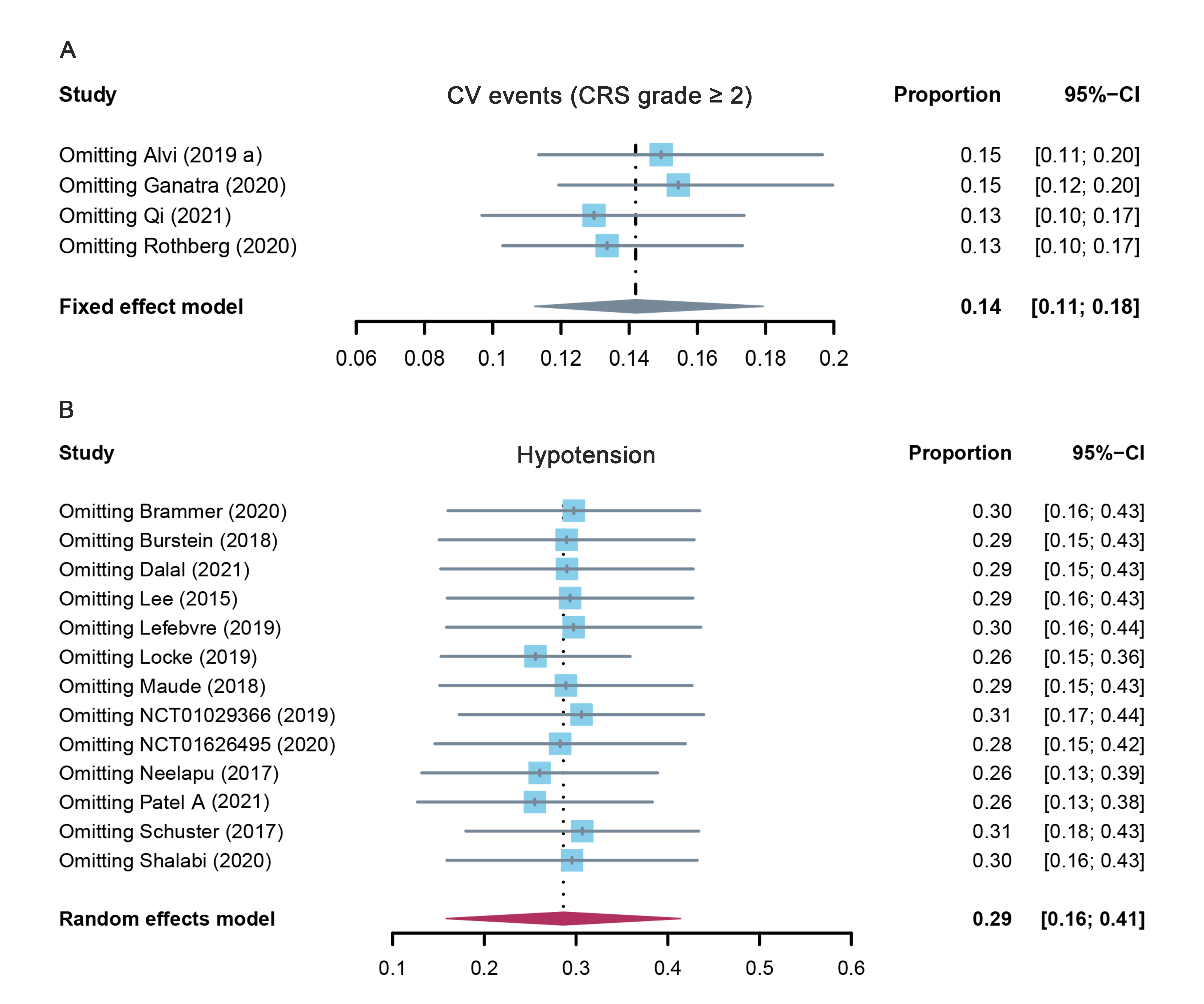

Supplement: Supplementary file 1 [file DataSheet_1.zip › Supplemental materials/Supplemental Figure S6.tif]

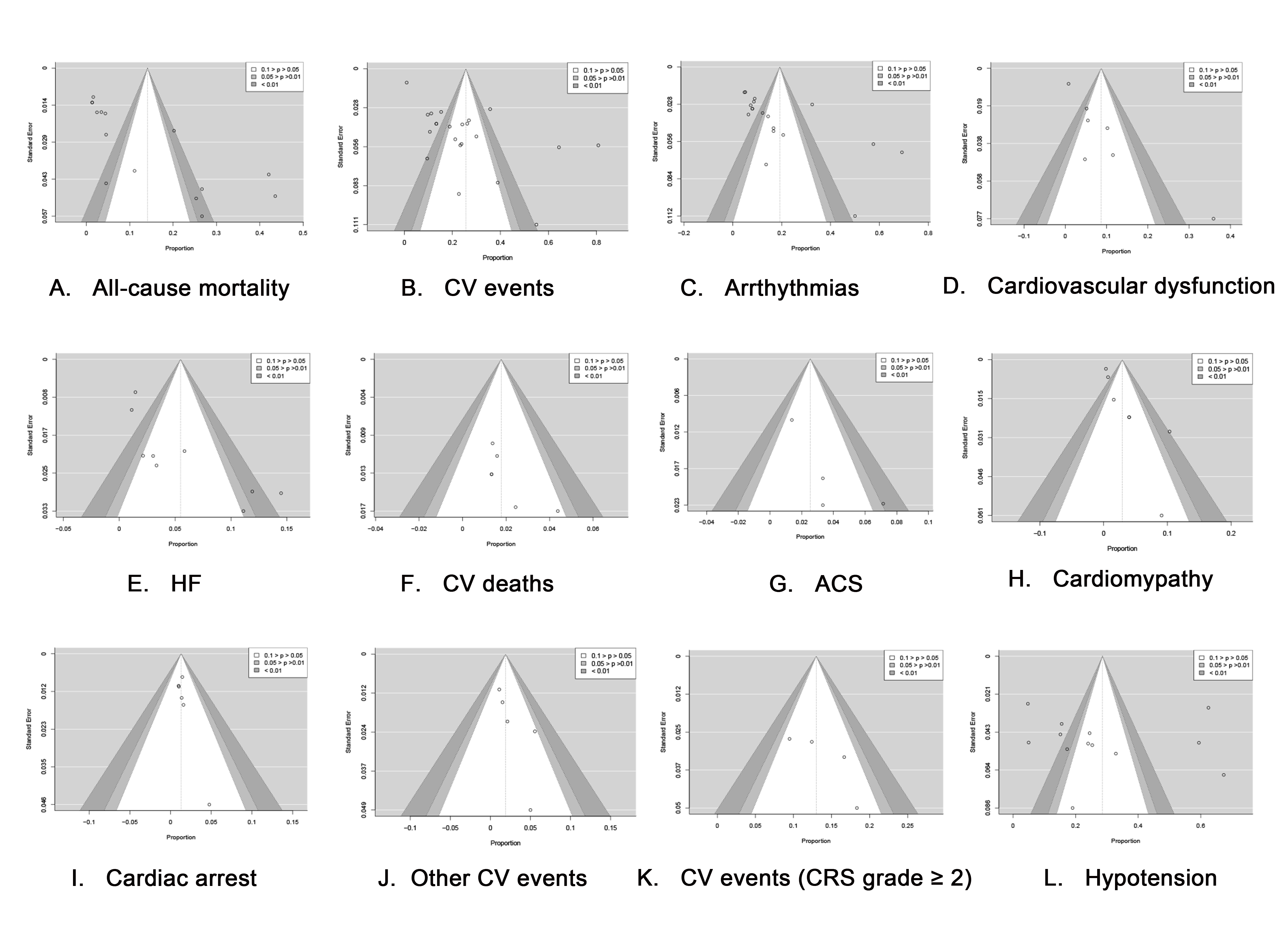

Supplement: Supplementary file 1 [file DataSheet_1.zip › Supplemental materials/Supplemental Figure S7.tif]
