# Supplementary material for: Cardiovascular effects associated with chimeric antigen receptor T cell therapy in cancer patients: A meta-analysis
Source: Front Oncol. 2022 Nov 9;12:924208. doi: 10.3389/fonc.2022.924208 (PMC9682079; doi:10.3389/fonc.2022.924208)
Supplement: Supplementary file 1 [file DataSheet_1.zip › Supplemental materials/1. Supplemental materials (Figure Legends).doc]

**Figure Legends**

**Supplemental Figure S1.** Risk of bias (RCT studies).

**Supplemental Figure S2.** The forest plot of pooled incidence of (A) CV deaths, (B) ACS, (C) cardiomyopathy, (D) cardiac arrest, and (E) other CV events incidence rate in patients who received CAR-T cell therapy. CV, cardiovascular; ACS, acute coronary syndrome; CAR-T cell, chimeric antigen receptor T cell.

**Supplemental Figure S3.** The sensitivity analysis of (A) CV deaths, (B) ACS, (C) cardiomyopathy, (D) cardiac arrest, and (E) other CV events incidence rate in patients who received CAR-T cell therapy. CV, cardiovascular; ACS, acute coronary syndrome; CAR-T cell, chimeric antigen receptor T cell.

**Supplemental Figure S4.** The forest plot of pooled incidence of (A) CV events (CRS grade ≥ 2) and (B) hypotension incidence rate in patients who received CAR-T cell therapy. CV, cardiovascular; CRS, cytokine release syndrome; CAR-T cell, chimeric antigen receptor T cell.

**Supplemental Figure S5.** The forest plot of pooled incidence rate of CRS among patients with CV events in patients who received CAR-T cell therapy. CRS, cytokine release syndrome; CV, cardiovascular; CAR-T cell, chimeric antigen receptor T cell.

**Supplemental Figure S6.** The sensitivity analysis of (A) CV events (CRS grade ≥ 2) and (B) hypotension incidence rate in patients who received CAR-T cell therapy. CV, cardiovascular; CRS, cytokine release syndrome; CAR-T cell, chimeric antigen receptor T cell.

**Supplemental Figure S7.** The funnel plot of (A) all-cause mortality, (B) CV events, (C) arrhythmias, (D) cardiovascular dysfunction, (E) HF, (F) CV deaths, (G) ACS, (H) cardiomyopathy, (I) cardiac arrest, (J) other CV events, (K) CV events (CRS grade ≥ 2), and (L) hypotension incidence rate in patients who received CAR-T cell therapy. CV, cardiovascular; HF, heart failure; ACS, acute coronary syndrome; CRS, cytokine release syndrome; CAR-T cell, chimeric antigen receptor T cell.
