# Supplementary material for: Cardiovascular effects associated with chimeric antigen receptor T cell therapy in cancer patients: A meta-analysis
Source: Front Oncol. 2022 Nov 9;12:924208. doi: 10.3389/fonc.2022.924208 (PMC9682079; doi:10.3389/fonc.2022.924208)
Supplement: Supplementary file 1 [file DataSheet_1.zip › Supplemental materials/Supplemental Table S1.doc]

**Supplementary Table S1:** The detailed example of the full electronic search strategy for PubMed.

| Search | Query | Items found |
| --- | --- | --- |
| 1 | ((Chimeric Antigen Receptor T-cell) OR (CAR T)) OR (CAR-T) | 9150 |
| 2 | (((Chimeric Antigen Receptor T-cell) OR (CAR T)) OR (CAR-T)) AND (((((cardiovascular toxicities) OR (cardiotoxicity)) OR (cardiovascular)) OR (cardiac)) OR (vascular)) | 321 |
| 3 | (((Chimeric Antigen Receptor T-cell) OR (CAR T)) OR (CAR-T)) AND ((((((((hematologic malignancies) OR (cancer)) OR (tumor)) OR (Neoplasm)) OR (Malignancies)) OR (Malignant Neoplasm)) OR (Benign Neoplasm)) OR (solid tumor)) | 7850 |
| 4 | ((((Chimeric Antigen Receptor T-cell) OR (CAR T)) OR (CAR-T)) AND ((((((((hematologic malignancies) OR (cancer)) OR (tumor)) OR (Neoplasm)) OR (Malignancies)) OR (Malignant Neoplasm)) OR (Benign Neoplasm)) OR (solid tumor))) AND ((((((cardiovascular toxicities) OR (cardiotoxicity)) OR (cardiovascular)) OR (cardiac)) OR (vascular)) OR (vascular)) | 243 |
