# Supplementary material for: Cardiovascular effects associated with chimeric antigen receptor T cell therapy in cancer patients: A meta-analysis
Source: Front Oncol. 2022 Nov 9;12:924208. doi: 10.3389/fonc.2022.924208 (PMC9682079; doi:10.3389/fonc.2022.924208)
Supplement: Supplementary file 1 [file DataSheet_1.zip › Supplemental materials/Supplemental Table S2.doc]

**Supplemental Table S2:** The quality assessment of included studies.

| **Study** | **Selection** | | | | | | **Comparability** | | | **Outcome** | | | | | **Quality**  **score** | |
| --- | --- | --- | --- | --- | --- | --- | --- | --- | --- | --- | --- | --- | --- | --- | --- | --- |
|  | representativeness of the exposed cohort | selection of the nonexposed cohort | Ascertainment of exposure | | Demonstration that outcome of interest was not present at start of study | Comparability of cohorts on the basis of the design or analysis | | Assessment of outcome | | | | Was follow-up long enough for outcome to occur | | Adequacy of follow up of cohorts |  |  |
| Alvi (2019a) | 1 | 0 | 1 | 1 | | 1 | | | 1 | | 1 | | 1 | | 7 |  |
| Alvi (2019b) | 1 | 0 | 1 | 1 | | 1 | | | 1 | | 0 | | 1 | | 6 |  |
| Brammer (2020) | 1 | 0 | 1 | 1 | | 1 | | | 1 | | 1 | | 1 | | 7 |  |
| Brammer (2021) | 1 | 0 | 1 | 1 | | 2 | | | 1 | | 1 | | 1 | | 8 |  |
| Burstein (2018) | 1 | 0 | 1 | 1 | | 2 | | | 1 | | 1 | | 1 | | 8 |  |
| Dalal (2021) | 1 | 0 | 0 | 1 | | 1 | | | 1 | | 1 | | 1 | | 6 |  |
| Ganatra (2020) | 1 | 0 | 1 | 1 | | 1 | | | 1 | | 1 | | 1 | | 7 |  |
| Lee (2021) | 0 | 0 | 1 | 1 | | 0 | | | 1 | | 1 | | 1 | | 5 |  |
| Lee (2022) | 0 | 0 | 1 | 1 | | 2 | | | 1 | | 0 | | 1 | | 6 |  |
| Lefebvre (2019) | 1 | 0 | 1 | 1 | | 1 | | | 1 | | 1 | | 1 | | 7 |  |
| Lefebvre (2020) | 1 | 0 | 1 | 0 | | 1 | | | 1 | | 1 | | 1 | | 6 |  |
| Locke (2019) | 1 | 0 | 1 | 1 | | 2 | | | 1 | | 1 | | 1 | | 8 |  |
| Patel A (2021) | 1 | 0 | 1 | 1 | | 1 | | | 1 | | 0 | | 1 | | 6 |  |
| Patel N (2021) | 1 | 0 | 1 | 1 | | 2 | | | 1 | | 1 | | 1 | | 8 |  |
| Qi (2021) | 1 | 0 | 1 | 0 | | 1 | | | 1 | | 0 | | 1 | | 5 |  |
| Rothberg (2020) | 1 | 0 | 1 | 1 | | 1 | | | 1 | | 1 | | 1 | | 7 |  |
| Shalabi (2020) | 1 | 0 | 1 | 1 | | 1 | | | 1 | | 0 | | 1 | | 6 |  |
| Wudhikarn (2020) | 1 | 0 | 1 | 1 | | 1 | | | 1 | | 1 | | 1 | | 7 |  |
