# Supplementary material for: Cardiovascular effects associated with chimeric antigen receptor T cell therapy in cancer patients: A meta-analysis
Source: Front Oncol. 2022 Nov 9;12:924208. doi: 10.3389/fonc.2022.924208 (PMC9682079; doi:10.3389/fonc.2022.924208)
Supplement: Supplementary file 1 [file DataSheet_1.zip › Supplemental materials/Supplemental Table S3.doc]

| **Supplemental Table S3:** The results of performed meta-analysis in subgroups of CAR-T target. | | | | | |
| --- | --- | --- | --- | --- | --- |
| Outcomes | Target antigen | No. of studies | Patients (n/N) | Pooled proportion (%) | 95%CI |
| All-cause mortality | | | | | |
|  | CD19 | 8 | 210/903 | 21.7 | [0.109; 0.325] |
|  | BCMA | 1 | 1/22 | 4.5 | [0.001; 0.228] |
|  | Others | 6 | 36/585 | 5.5 | [0.000; 0.123] |
| CV events | | | | | |
|  | CD19 | 12 | 286/1063 | 26.0 | [0.156; 0.365] |
|  | BCMA | 1 | 5/22 | 22.7 | [0.078; 0.454] |
|  | Others | 9 | 182/787 | 25.4 | [0.115; 0.394] |
| Arrhythmias | | | | | |
|  | CD19 | 8 | 192/807 | 24.1 | [0.110; 0.372] |
|  | BCMA | 1 | 3/22 | 13.6 | [0.029; 0.349] |
|  | Others | 9 | 101/787 | 19.2 | [0.107; 0.277] |
| Cardiovascular dysfunction | | | | | |
|  | CD19 | 5 | 35/347 | 9.8 | [0.043; 0.225] |
|  | Others | 2 | 7/178 | 3.5 | [0.003; 0.479] |
| HF | | | | | |
|  | CD19 | 4 | 28/535 | 3.9 | [0.012; 0.125] |
|  | Others | 5 | 36/503 | 7.1 | [0.040; 0.126] |
| CV deaths | | | | | |
|  | CD19 | 1 | 2/145 | 1.4 | [0.002; 0.049] |
|  | Others | 5 | 12/495 | 1.9 | [0.007; 0.032] |
| ACS | | | | | |
|  | CD19 | 1 | 2/145 | 1.4 | [0.002; 0.049] |
|  | Others | 3 | 14/276 | 4.4 | [0.020; 0.069] |

**Supplemental Table S3. Cont.**

| Outcomes | Target antigen | No. of studies | Patients (n/N) | Pooled proportion (%) | 95%CI |
| --- | --- | --- | --- | --- | --- |
| Cardiomyopathy | | | | | |
|  | CD19 | 4 | 15/601 | 2.5 | [0.000; 0.063] |
|  | BCMA | 1 | 2/22 | 9.1 | [0.011; 0.292] |
|  | Others | 2 | 6/150 | 4.0 | [0.009; 0.071] |
| Cardiac arrest | | | | | |
|  | CD19 | 6 | 9/633 | 1.3 | [0.004; 0.022] |
| Other CV events | | | | | |
|  | CD19 | 3 | 3/133 | 1.9 | [0.000; 0.042] |
|  | Others | 2 | 6/180 | 2.8 | [0.000; 0.071] |
| CV events (CRS grade ≥ 2) | | | | | |
|  | CD19 | 1 | 11/116 | 9.5 | [0.048; 0.163] |
|  | Others | 3 | 49/323 | 15.4 | [0.119; 0.200] |
| Hypotension | | | | | |
|  | CD19 | 10 | 351/843 | 31.5 | [0.169; 0.461] |
|  | Others | 3 | 41/217 | 18.4 | [0.132; 0.235] |

**Abbreviations:** CAR-T, chimeric antigen receptor T; CV, cardiovascular events; HF, heart failure; ACS, acute coronary syndrome; CRS, cytokine release syndrome.
