# Supplementary material for: Cardiovascular effects associated with chimeric antigen receptor T cell therapy in cancer patients: A meta-analysis
Source: Front Oncol. 2022 Nov 9;12:924208. doi: 10.3389/fonc.2022.924208 (PMC9682079; doi:10.3389/fonc.2022.924208)
Supplement: Supplementary file 1 [file DataSheet_1.zip › Supplemental materials/Supplemental Table S4.doc]

| **Supplemental Table S4:** The pooled proportions of outcomes for cancer with CAR-T cell therapy. | | | | | | | |
| --- | --- | --- | --- | --- | --- | --- | --- |
| Outcomes | No. of studies | Patients (n/N) | Pooled proportion (%) | 95%CI | Heterogeneity within study | | Publication bias |
|  |  |  |  |  | I^2^(%) | p-value | p-value |
| All-cause mortality | 15 | 247/1510 | 14.1% | [0.067; 0.216] | 95% | < 0.01 | < 0.05 |
| CV events | 22 | 473/1872 | 25.6% | [0.177; 0.335] | 96% | < 0.01 | < 0.05 |
| Arrhythmias | 18 | 296/1616 | 19.2% | [0.107; 0.277] | 93% | < 0.01 | <0.05 |
| Cardiovascular dysfunction | 7 | 42/525 | 8.0% | [0.031; 0.129] | 84% | < 0.01 | <0.05 |
| HF | 9 | 64/1038 | 5.3% | [0.026; 0.079] | 80% | < 0.01 | <0.05 |
| CV deaths | 6 | 14/640 | 1.8% | [0.008; 0.028] | 0% | =0.73 | >0.05 |
| ACS | 4 | 16/421 | 2.5% | [0.010; 0.040] | 48% | = 0.12 | <0.05 |
| Cardiomyopathy | 7 | 23/773 | 2.9% | [0.006; 0.054] | 68% | < 0.01 | <0.05 |
| Cardiac arrest | 6 | 9/633 | 1.3% | [0.004; 0.022] | 0% | = 0.98 | >0.05 |
| Other CV events | 5 | 9/313 | 1.9% | [0.004; 0.034] | 0% | = 0.51 | >0.05 |
| CV events (CRS grade ≥ 2) | 4 | 60/439 | 14.2% | [0.112; 0.179] | 22% | = 0.28 | >0.05 |
| Hypotension | 13 | 392/1060 | 28.6% | [0.158; 0.414] | 96% | < 0.01 | >0.05 |

**Abbreviations:** CAR-T cell, chimeric antigen receptor T cell; CV, cardiovascular events; HF, heart failure; ACS, acute coronary syndrome; CRS, cytokine release syndrome.
